# Prognostic significance of esterase gene expression in multiple myeloma

**DOI:** 10.1038/s41416-020-01237-1

**Published:** 2021-02-03

**Authors:** Romika Kumari, Muntasir Mamun Majumder, Juha Lievonen, Raija Silvennoinen, Pekka Anttila, Nina N. Nupponen, Fredrik Lehmann, Caroline A. Heckman

**Affiliations:** 1 Institute for Molecular Medicine Finland (FIMM), Helsinki, Finland; 2 HiLIFE–Helsinki Institute of Life Science, University of Helsinki, Helsinki, Finland; 3 iCAN–Digital Precision Cancer Medicine Flagship, Helsinki, Finland; 4 Department of Hematology, Helsinki University Hospital Comprehensive Cancer Center, and University of Helsinki, Helsinki, Finland; 5 Oncopeptides AB, Stockholm, Sweden

**Keywords:** Tumour biomarkers, Tumour biomarkers

## Abstract

**Background:**

Esterase enzymes differ in substrate specificity and biological function and may display dysregulated expression in cancer. This study evaluated the biological significance of esterase expression in multiple myeloma (MM).

**Methods:**

For gene expression profiling and evaluation of genomic variants in the Institute for Molecular Medicine Finland (FIMM) cohort, bone marrow aspirates were obtained from patients with newly diagnosed MM (NDMM) or relapsed/refractory MM (RRMM). CD138+ plasma cells were enriched and used for RNA sequencing and analysis, and to evaluate genomic variation. The Multiple Myeloma Research Foundation (MMRF) Relating Clinical Outcomes in MM to Personal Assessment of Genetic Profile (CoMMpass) dataset was used for validation of the findings from FIMM.

**Results:**

MM patients (NDMM, *n* = 56; RRMM, *n* = 78) provided 171 bone marrow aspirates (NDMM, *n* = 56; RRMM, *n* = 115). Specific esterases exhibited relatively high or low expression in MM, and expression of specific esterases (*UCHL5*, *SIAE*, *ESD*, *PAFAH1B3*, *PNPLA4* and *PON1*) was significantly altered on progression from NDMM to RRMM. High expression of *OVCA2*, *PAFAH1B3*, *SIAE* and *USP4*, and low expression of *PCED1B*, were identified as poor prognostic markers (*P* < 0.05). The MMRF CoMMpass dataset provided validation that higher expression of *PAFAH1B3* and *SIAE*, and lower expression of *PCED1B*, were associated with poor prognosis.

**Conclusions:**

Esterase gene expression levels change as patients progress from NDMM to RRMM. High expression of *OVCA2*, *PAFAH1B3*, *USP4* and *SIAE*, and low expression of *PCED1B*, are poor prognostic markers in MM, suggesting a role for these esterases in myeloma biology.

## Background

Both antibody–drug conjugates (ADCs) and peptide–drug conjugates (PDCs) represent important therapeutic classes that enable the selective introduction of cytotoxic drugs into cancer cells over healthy cells, potentially improving efficacy and reducing systemic toxicity compared with non-conjugated versions of the same drug.^1,2^ Conceptual similarities between ADCs and PDCs include selective targeting and subsequent cellular internalisation, although the exact mechanism of action is specific to each individual conjugate. To release cytotoxic payloads within the target cell, ADCs are typically endocytosed and cleaved within the consequent lysosomal structure, whereas PDCs undergo hydrolytic cleavage in the cell via interactions with cell surface receptors, or after direct entry into the cytosol through the cell membrane.^1–3^ Various different enzyme classes may be involved in conjugate metabolism and release of the cytotoxic payload in ADCs and PDCs, and hence research interest in metabolising enzymes is growing for both therapeutic approaches.^1–3^

The esterase enzyme family is a subclass of the hydrolase enzyme superfamily that functions to hydrolyse ester bonds.^4,5^ Many different esterases have been identified, which differ in their substrate specificity and biological function, and the expression of specific esterases may be dysregulated in cancer. Hydrolysing enzymes can be highly expressed in cancer cells, and have previously been implicated in the reprogramming of metabolic pathways, promotion of cancer pathogenesis, drug metabolism and drug toxicity.^4,5^ Esterases expressed in tumour cells may also differ in their stereoselectivity for hydrolysis of chiral esters compared with esterases expressed in healthy tissues.^6–9^ Esterases can themselves also be administered to treat haematological malignancies; for example, the enzyme asparaginase has been used as an effective agent to treat acute lymphoblastic leukaemia for many years,^10^ and it is also being investigated for the treatment of acute myeloid leukaemia.^11^

Esterase hydrolysis, therefore, represents an interesting potential strategy for selective activation of anticancer drugs within cancer cells that overexpress esterases, or which express esterases with different specificities, while minimising toxic effects on healthy cells and tissues.^6^ The study of esterase expression in cancer is still in its infancy, but interest is growing as a result of the development of novel PDCs such as melflufen (melphalan flufenamide), which utilises intracellular aminopeptidases and esterases to release a cytotoxic payload in multiple myeloma cells.^3,12^

Several esterases have previously been reported to be dysregulated in specific cancers, with some also having the potential to be predictive or prognostic biomarkers. For example, low expression of neurexophilin and PC-esterase domain family member 4 (*NXPE4*) mRNA is a prognostic marker for shorter survival in patients with colorectal cancer,^13^ and expression of platelet-activating factor acetylhydrolase 1B2 (*PAFAH1B2*) is inversely correlated with patient survival in pancreatic ductal adenocarcinoma.^14^ In lung cancer, acetylcholinesterase (*ACHE*) may act as a tumour suppressor and is downregulated,^15^ and high expression of granzyme A (*GZMA*) is significantly associated with an improved prognosis in multiple cancer types.^16^ Sequencing studies have identified a number of genes that are mutated with high frequency in multiple myeloma,^17,18^ although genes encoding esterases do not feature prominently among them. This is most likely because esterase genes do not have high-frequency mutation rates in multiple myeloma, which in turn suggests that regulation of esterase gene expression occurs through mechanisms other than mutations. Still, expression of esterase genes can be dysregulated in this disease: carboxylesterase 1 (*CES1*), *CES2* and butyrylcholinesterase (*BCHE*) can both be overexpressed in multiple myeloma cells,^19,20^ and *BCHE* is associated with a poor prognosis in multiple myeloma and dichotomously expressed, suggesting its tightly controlled regulation.^21^ Also, expression of ubiquitin carboxyl-terminal esterase L1 (*UCHL1*) in samples from patients with newly diagnosed multiple myeloma (NDMM) correlated with a high-risk subgroup of myeloma.^22^ Other studies have suggested possible mechanisms for the role of esterases in the pathogenesis of multiple myeloma; these include impaired oxidative/antioxidative balance due to reduced activity of serum paraoxonase-1 and arylesterase,^23^ and inhibition of ubiquitin-specific protease 14 and ubiquitin-C-terminal hydrolase-5 (UCHL5).^24,25^

In this study, we evaluated the biological significance of a panel of esterases in multiple myeloma using gene expression profiling, cytogenetic profiling and clinical outcome analyses. We demonstrate that specific esterases exhibit relatively high or low expression in multiple myeloma, that the esterase expression profile changes on the progression of the disease, and that high or low expression of individual esterases is associated with poor prognosis. Although single-nucleotide variants (SNVs) were rare in the esterase genes, several had duplication and deletion copy number alterations.

## Methods

### Sample collection and plasma cell enrichment

For gene expression profiling and evaluation of genomic variants in the Institute for Molecular Medicine Finland (FIMM) cohort, bone marrow aspirates were obtained from multiple myeloma patients after obtaining written informed consent and following protocols approved by an ethical committee of the Helsinki University Hospital Comprehensive Cancer Center, and in compliance with the Declaration of Helsinki. Matched patient skin biopsies were collected (also with informed consent) at the same time and from the same site as bone marrow aspirates, and in accordance with approved protocols, for constitutional DNA analysis. Bone marrow mononuclear cells were isolated by Ficoll-Paque gradient centrifugation (GE Healthcare), and CD138+ plasma cells enriched by immuno-magnetic bead selection (StemCell Technologies).

### RNA sequencing and analysis

RNA was extracted from CD138+ plasma cells using the AllPrep^®^ DNA/RNA/miRNA Universal or miRNeasy kits (Qiagen). RNA integrity was measured on an Agilent Bioanalyzer 2100 instrument; only samples with RNA integrity ≥7 were used for sequencing. Illumina-compatible RNA sequencing libraries were prepared using Scriptseq™ or Nextera technology and sequenced on Illumina HiSeq^®^ 1500 or 2500 instruments (Illumina). After pre-processing, filtered reads were aligned to the GRCh38 human reference genome using the STAR aligner tool.^26^ Gene read counts were normalised using the Reads Per Kilobase of transcript per million mapped reads (RPKM) method. In total, 51 annotated esterase genes (Supplementary Table S1) were identified in the human genome (assembly GRCh38) utilising the Ensembl release 99^27^ and NCBI^28^ databases, using the search term ‘esterase’ and further confirming the molecular function (gene ontology) of identified genes. A cut-off value of >1 RPKM was used to filter expressed esterase genes. DEseq2^29^ was used to identify variation in gene expression in newly diagnosed versus relapsed/refractory samples. The contribution of esterase gene expression to survival outcome was estimated by Kaplan–Meier analysis and performed using expression-based filtering. For each esterase gene, samples were stratified into ‘high’ (≥median expression) and ‘low’ (<median expression) expression groups. The significance of the difference between the two groups (high versus low expression) was deduced using a Mantel–Cox log-rank test.

### Exome sequencing and cytogenetics

The DNeasy^®^ Blood & Tissue kit or AllPrep^®^ DNA/RNA/miRNA Universal kit (Qiagen) was used to isolate genomic DNA from skin biopsies and CD138+ cells. The SeqCap^®^ EZ MedExome kit (Roche NimbleGen), SureSelect Clinical Research Exome kit or SureSelect Human All Exon V5 kit (Agilent Technologies) was used for exome capture. Sequencing was performed on HiSeq^®^ 1500 or 2500 instruments. VarScan2 somatic algorithm^30^ was implemented for calling somatic mutations, and mutation annotations were performed using SnpEff 4.04^31^ as described previously.^32^ Gene copy number variants (CNVs) were identified using the CopyCat tool (https://github.com/chrisamiller/copycat). Cytogenetics data were generated using fluorescence in situ hybridisation technology as described previously,^33^ following European Myeloma Network 2012 guidelines.^34^

### Data validation

To validate our results, clinical, gene expression and genomic variant data (somatic mutation and CNVs) were obtained from the Multiple Myeloma Research Foundation (MMRF) Relating Clinical Outcomes in MM to Personal Assessment of Genetic Profile (CoMMpass) study (https://research.themmrf.org, www.themmrf.org). The MMRF CoMMpass gene expression dataset includes 892 samples: 87% baseline/diagnosis, 12% progressive disease and 1% missing. See Supplementary Material for further details.

## Results

### Patient population

In total, 134 patients provided 171 bone marrow aspirates for the FIMM dataset: 56 samples for the NDMM subgroup and 115 samples for the relapsed/refractory multiple myeloma (RRMM) subgroup (see Supplementary Fig. S1 for a patient/sample flow chart). Individual patients could provide samples only at diagnosis (*n* = 49), at both diagnosis and relapse (*n* = 7), only at first relapse (*n* = 59) or at recurring relapses, i.e., multiple relapse samples from one patient (second relapse, *n* = 13; third relapse, *n* = 3; fourth relapse, *n* = 2; sixth relapse, *n* = 1). The median age was similar in the NDMM and RRMM subgroups, and there was a higher proportion of males (78/134, 58.2%) in the total population (Table 1). More patients in the RRMM subgroup (*n* = 20, 25.6%) had a 17p deletion compared with the NDMM subgroup (*n* = 5, 8.9%; Fisher exact test *P* = 0.015). Chromosome 1q gain was also more common in the RRMM subgroup (*n* = 44, 56.4%) compared with the NDMM group (*n* = 14, 25.0%; Fisher exact test *P* = 0.0004). In the RRMM subgroup, previous treatments included alkylating agents in 97.4%, bortezomib in 88.5% and immunomodulatory drugs in 79.5% of patients.

**Table 1:** Patient characteristics, disease characteristics and prior therapies in patients with multiple myeloma in the in-house FIMM cohort.

Patient and disease characteristics by disease stage^a^			
	NDMM (*n* = 56)	RRMM (*n* = 78)	Total (*N* = 134)
Age at diagnosis, years, median (range)	64.5 (26–84)	63 (41–81)	64 (26–84)
Sex, female/male, *n*	26/30	30/48	56/78
*Cytogenetics,* *n* *(%)*			
t(11;14)	16 (28.6)	14 (17.9)	30 (22.4)
t(4;14)	7 (12.5)	18 (23.1)	25 (18.7)
t(14;16)	1 (1.8)	2 (2.6)	3 (2.2)
t(14;20)	0	2 (2.6)	2 (1.5)
del(17p)	5 (8.9)	20 (25.6)	25 (18.7)
del(13q)	36 (64.3)	40 (51.3)	76 (56.7)
1q gain	14 (25.0)	44 (56.4)	58 (43.3)
Missing	0	2 (2.6)	2 (1.5)
*ISS,* *n* *(%)*			
1	13 (23.2)	16 (20.5)	29 (21.6)
2	26 (46.4)	22 (28.2)	48 (35.8)
3	10 (17.9)	16 (20.5)	26 (19.4)
Missing	7 (12.5)	24 (30.8)	31 (23.1)
**Treatment history of relapsed/refractory patients (*N*** **= 78)**			
	**Exposed, relapsed**	**Exposed, refractory**	**Not exposed**
Prior treatment, *n* (%)			
Alkylating agents (MEL, CPM)	61 (78.2)	15 (19.2)	2 (2.6)
Bortezomib	43 (55.1)	26 (33.3)	9 (11.5)
IMiDs	29 (37.2)	33 (42.3)	16 (20.5)

*CPM* cyclophosphamide, *FIMM* Institute for Molecular Medicine Finland, *IMiD* immunomodulatory imide drug, *ISS* International Staging System, *MEL* melphalan, *NDMM* newly diagnosed multiple myeloma, *RRMM* relapsed/refractory multiple myeloma.

^a^If a patient provided both NDMM and RRMM samples, this patient was included in the NDMM group. If a patient provided samples at multiple relapse stages and the diagnosis sample is missing, then data from the first relapse are included in the table.

### Esterase gene expression profile in multiple myeloma samples

RNA extracted from 123 of 171 CD138+ plasma cell patient samples was suitable for RNA sequencing analysis, which included 41 samples from patients with NDMM, and 82 samples from patients with RRMM.

**Fig. 1:**
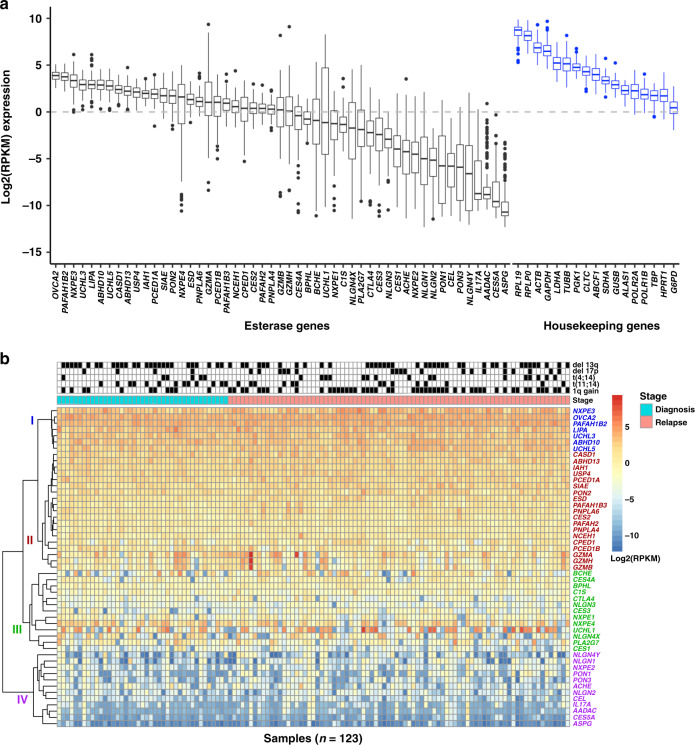
Esterase gene expression profile in multiple myeloma samples from the in-house FIMM dataset. **a** Log2(RPKM) expression of esterases and housekeeping genes, ranked based on median expression values.^a^ **b** Esterase expression heatmap and hierarchical clustering based on expression level^b^. FIMM Institute for Molecular Medicine Finland, MM multiple myeloma, RPKM reads per kilobase of transcript per million mapped reads. *n* = 123 samples; genes with log2(RPKM) > 0 were considered as expressed. ^a^Box plots: thick central line represents median; top and bottom lines of box represent third quartile and first quartile; whiskers indicate the variability in the data outside the upper and lower quartile; filled black circles represent outliers. ^b^The unsupervised hierarchical clustering of esterase gene expression profiles was performed using method complete-linkage and Manhattan distance measures.

Esterase gene expression levels were ranked based on their abundance (Fig. 1a and Supplementary Table S1). The most abundant esterase mRNAs were ovarian tumour suppressor candidate 2 (*OVCA2*), *PAFAH1B2*, *NXPE3*, *UCHL3*, lipase A lysosomal acid type (*LIPA*), abhydrolase domain containing 10 (*ABHD10*), *UCHL5*, N-acetylneuraminate 9-O-acetyltransferase (*CASD1*), *ABHD13* and ubiquitin carboxyl-terminal hydrolase 4 (*USP4*), with a median log2[RPKM] range of 3.9 (*OVCA2*) to 2.1 (*USP4*). The least abundant esterases were asparaginase (*ASPG*), *CES5A*, arylacetamide deacetylase (*AADAC*), interleukin-17A (*IL17A*), neuroligin 4 Y-linked (*NLGN4Y*), paraoxonase 3 (*PON3*), cholesterol esterase (*CEL*), *PON1*, neuroligin-2 (*NLGN2*) and *NLGN1*, with a median log2[RPKM] range of −10.7 (*ASPG*) to −5.1 (*NLGN1*; Fig. 1a). Similar esterase mRNA expression patterns were observed (*r* = 0.9, *P* value = 2.2e-16) utilising the MMRF CoMMpass dataset (*N* = 892) (Supplementary Figs. S2, S3). In our FIMM dataset, the esterase genes clustered in four subgroups based on expression level, with group I having the highest and group IV having the lowest level of expression (Fig. 1b).

**Fig. 2:**
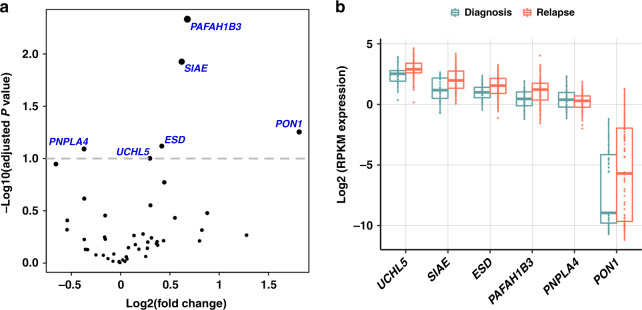
Esterase genes are differentially expressed in newly diagnosed multiple myeloma (*n* = 41) versus relapsed/refractory multiple myeloma (*n* = 82) samples in the in-house FIMM dataset. **a** DEseq2 differential expression results for 51 esterase genes. **b** The expression values of genes (*n* = 6) predicted to be differentially regulated in NDMM versus RRMM groups^a^. FIMM Institute for Molecular Medicine Finland, NDMM newly diagnosed multiple myeloma, RPKM reads per kilobase of transcript per million mapped reads, RRMM relapsed/refractory multiple myeloma. ^a^Box plots: thick central line represents median; top and bottom lines of box represent third quartile and first quartile; whiskers indicate the variability in the data outside the upper and lower quartile; circles inside the boxplot/distribution represent data point locations; circles outside the boxplot/distribution represent outliers.

The mRNA expression profiles of several esterases were significantly different in samples from newly diagnosed patients versus those whose disease had relapsed or were refractory to treatment (Fig. 2). The expression levels of *UCHL5*, sialic acid acetyl esterase (*SIAE)*, esterase D (*ESD)*, *PAFAH1B3* and *PON1* were significantly higher (*P* ≤ 0.01; adjusted *P* < 0.1) in RRMM versus NDMM samples, and the expression level of PNPLA4 was significantly lower (*P* = 0.007). In the MMRF CoMMpass dataset using paired samples (NDMM, *n* = 39; RRMM, *n* = 45), we observed similar expression patterns, with the median expression of the majority of these esterase genes being higher in RRMM samples. Whereas gene *PNPLA4* had a conflicting expression pattern with expression being higher in RRMM samples in the CoMMpass dataset. However, none of the genes were found to have *P* values  ≤ 0.05 (Supplementary Fig. S4) in the DEseq2 analysis. In addition, the expression of the esterase gene phospholipase A2 Group VII (*PLA2G7*; *P* < 0.001) was significantly different in NDMM versus RRMM samples using paired samples from the MMRF CoMMpass data (data not shown).

DEseq2 analysis also revealed that out of all the esterases (*n* = 6) predicted to be differentially regulated in NDMM versus RRMM samples in our dataset, only *UCHL5* was upregulated in both RRMM samples as well as samples with 1q gain (Supplementary Fig. S5).

### Prognostic significance of esterase expression in multiple myeloma

**Fig. 3:**
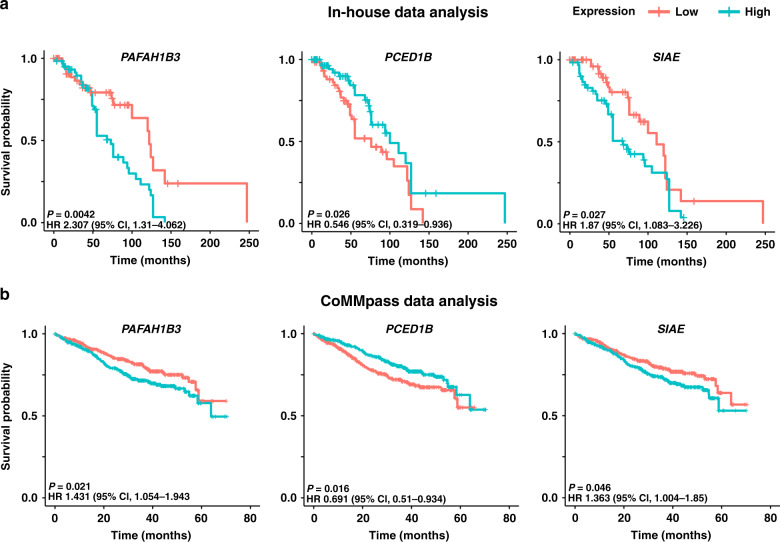
Prognostic significance of esterase expression. Low expression of *PCED1B* and high expression of *PAFAH1B3 and SIAE* are associated with poor prognosis in both (**a**) the in-house FIMM dataset and (**b**) the MMRF CoMMpass validation dataset. CL confidence limit, CoMMpass relating clinical outcomes in MM to personal assessment of genetic profile, FIMM Institute for Molecular Medicine Finland, HR hazard ratio, MMRF Multiple Myeloma Research Foundation.

In our FIMM dataset, patient samples exhibiting high expression of *OVCA2*, *PAFAH1B3*, *SIAE* and *USP4* were associated with a significantly poorer prognosis versus those with low expression (Fig. 3a, Supplementary Figs S6, S7 and Supplementary Table S2); median overall survival for high versus low expression samples was 68 versus 122 months for *OVCA2* (hazard ratio (HR) 3.35, 95% confidence limit (CL) 1.811–6.198; *P* < 0.0001), 73 versus 122 months for *PAFAH1B3* (HR 2.307, 95% CL 1.31–4.062; *P* = 0.0042), 68 versus 111 months for *SIAE* (HR 1.87, 95% CL 1.083–3.226; *P* = 0.027) and 74 versus 122 months for *USP4* (HR 1.76, 95% CL 1.012–3.059; *P* = 0.041). Genes with an overall expression of <1 RPKM were not included in this analysis. Expression of *OVCA2* was significantly lower (Welch’s *t* test, *P* = 0.003) in samples with del17p (*n* = 19) than in those without (*n* = 104; Supplementary Fig. S8).

Patient samples exhibiting low expression of *GZMA, PCED1B* and *NXPE3* were associated with poorer prognosis versus those with high expression (Fig. 3a, Supplementary Figs. S6, S7 and Supplementary Table S2); median overall survival for high versus low expression samples was 120 versus 55 months for *GZMA* (HR 0.517, 95% CL 0.306–0.874; *P* = 0.013), 100 versus 76 months for *PCED1B* (HR 0.546, 95% CL 0.319–0.936; *P* = 0.026) and 96 versus 76 months for *NXPE3* (HR 0.578, 95% CL 0.337–0.991). A visual comparison of the *P* values for all data is shown (Supplementary Fig. S6).

The MMRF CoMMpass dataset provided validation that higher expression of *PAFAH1B3* (HR 1.431; 95% CL 1.054–1.943; *P* = 0.021) and *SIAE* (HR 1.363; 95% CL 1.004–1.85; *P* = 0.046), and lower expression of *PCED1B* (HR 0.691; 95% CL 0.51–0.934; *P* = 0.016) were associated with poor prognosis (Fig. 3b). Several other esterases (*NXPE4*, *UCHL5, PAFAH1B2*, *BPHL, NXPE1, UCHL3*) not identified in the FIMM dataset were predicted to have a role in disease prognosis in the MMRF CoMMpass dataset (Supplementary Fig. S6).

### Somatic mutation and copy number variation

Of 171 patient samples, 169 were successfully processed for exome sequencing, comprising 56 samples from patients with NDMM and 113 samples from patients with RRMM.

Exome sequencing data revealed that somatic SNVs are rare in esterase genes. In this cohort, somatic SNVs were observed in the *CEL* gene at a frequency of 3.5% (6/169 samples; no hotspot mutations, *n* = 6 non-synonymous) and in the *NXPE1* gene at a frequency of 2.3% (4/169 samples; no hotspot mutations, *n* = 4 non-synonymous), while the frequency of SNVs in other esterase genes was below 2% (*PCED1B* 0.59%, 1/169 samples; *PAFAH1B3* 0%; *SIAE* 0%; Supplementary Fig. S9). Mutation frequencies in esterase genes were also rare in the MMRF CoMMpass dataset, with all esterases being mutated in <2% of samples (*N* = 1164; Supplementary Fig. S10).

**Fig. 4:**
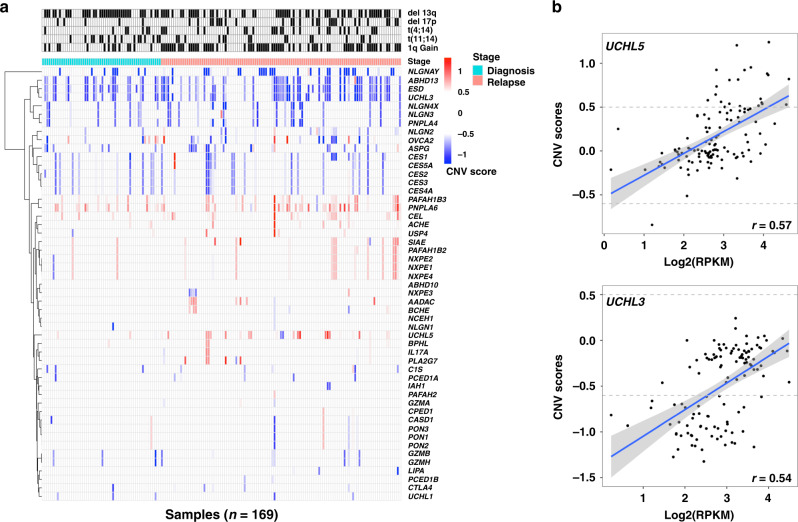
Multiple esterase genes have copy number alterations. **a** Heatmap showing clustering of esterase genes based on CNV scores.^a^ **b** Correlation comparison of gene expression and CNV scores for the genes *UCHL3* and *UCHL5*. *CNV* copy number variation. ^a^A CNV score of more than 0.5 predicts a duplication/gain event and a CNV score of less than −0.6 predicts a deletion event.

In contrast, multiple esterase genes were found to have copy number alterations of both duplication and deletion types (Fig. 4). *PNPLA6* was found to have a copy number gain in 30.8% (52/169) of samples, *PAFAH1B3* had a copy number gain in 18.9% (32/169) of samples and *CEL* had a copy number gain in 17.2% (29/169) of samples. Deletions in genes encoding *ESD*, *UCHL3* and *ABHD13* were observed in more than 38% of the samples (Fig. 4a). These results were validated in the MMRF CoMMpass dataset: *PNPLA6* was found to have a copy number gain in 40.1% (419/1044) of samples, *CEL* was found to have a copy number gain in 38.5% (402/1044) of samples and *PAFAH1B3* had a copy number gain in 31.7% (331/1044) of samples. Deletions in genes encoding *ESD*, *UCHL3* and *ABHD13* were observed in ~36–41% of samples (Supplementary Fig. S11). Furthermore, in the FIMM cohort, a correlation analysis of gene expression and CNVs revealed that among all genes predicted to have CNVs, only the genes *UCHL5* and *UCHL3* had a weak positive correlation (*r* > 0.5) of 0.57 and 0.54 with corresponding log2(RPKM) gene expression values (Fig. 4b).

## Discussion

A detailed understanding of the role of specific metabolic enzymes in the processing of novel therapeutic approaches such as ADCs and PDCs may be critical for their success in the treatment of specific cancers. The majority of chemical linkers in ADCs are based on hydrazone, disulfide, thioester or peptide bonds; these linkers are designed to exploit differences in intracellular pH, intracellular reduction potential or intracellular metabolic enzyme concentrations to break the linker and release a cytotoxic payload into tumour cells.^1^ The cytotoxic activity of PDCs is also dependent on intracellular metabolic enzyme concentrations.^2^ Therefore, among other enzymes, esterases have a significant potential for exploitation in drug design. However, their role in cancer, and more specifically in multiple myeloma, has not been widely studied to date, despite myeloma being a disease of altered protein homoeostasis that is commonly targeted with proteasome inhibitors. Novel lipophilic PDCs such as melflufen (melphalan flufenamide) can readily diffuse into multiple myeloma cells, where high expression of aminopeptidases results in hydrolytic cleavage and rapid release of the cytotoxic alkylator payload, and esterases can also create active intermediate metabolites.^3^ Therefore, the role of these enzymes in myeloma cells is of particular interest; for example, aminopeptidase and esterase gene expression profiles could, hypothetically, be used to identify patient subgroups who are more likely to respond to drugs such as melflufen, which utilise these enzymes as part of their mechanism of action.

In this study, we demonstrate for the first time that several individual esterase genes exhibit relatively high or low median expression in bone marrow aspirates from patients with multiple myeloma. Interestingly, the expression profile of several genes appeared to change on progression from NDMM to RRMM; the expression of *UCHL5*, sialic acid acetyl esterase (*SIAE)*, esterase D *(ESD)*, *PAFAH1B3* and *PON1* was significantly higher in RRMM versus NDMM samples, whereas the expression of *PNPLA4* was significantly lower in RRMM versus NDMM samples. Among these differentially expressed genes, only *UCHL5* was shown to be upregulated in samples with 1q gain (a common cytogenetic abnormality in MM), which is likely due to the genomic location of *UCHL5* (1q31.2). UCHL5 is a deubiquitylating enzyme (DUB) that is more highly expressed in MM cells than in normal plasma cells.^25^ Preclinical studies have shown that inhibition of UCHL5 decreases viability and inhibits proliferation of MM cells, and overcomes resistance to bortezomib.^25^ Many DUBs demonstrate esterase activity, and DUB inhibitors have been studied for anti-myeloma activity. One such inhibitor, VLX1570, has been assessed in a phase 1 study of patients with RRMM;^35^ this study was discontinued due to severe pulmonary toxicity. Nevertheless, further efforts to identify DUB inhibitors with a wider therapeutic index may be warranted given promising preclinical anti-tumour effects and activity in MM resistant to proteasome inhibitors.^25,35^

In our dataset, high expression of *OVCA2*, *PAFAH1B3*, *SIAE* and *USP4* was associated with a significantly poorer prognosis compared with samples showing low expression, whereas low expression of *GZMA, PCED1B and NXPE3* was associated with a significantly poorer prognosis compared with samples expressing higher levels of the enzyme. Esterase expression patterns were similar in the MMRF CoMMpass validation dataset, but of the genes identified as being prognostic in our dataset, only high expression of *PAFAH1B3 and SIAE*, and low expression of *PCED1B* was associated with poor prognosis in the CoMMpass dataset. Other esterases predicted to have a role in disease prognosis only in the validation dataset included *NXPE4*, *UCHL5, PAFAH1B2*, *BPHL*, *NXPE1* and *UCHL3*. The differences observed between datasets may simply have been caused by the lower number of samples in our dataset, or by technical differences in RNA sequencing library preparation methods. Another possible reason for the differences may have been that our dataset includes samples from patients with NDMM (32.7%) or RRMM (67.3%), whereas the CoMMpass dataset includes mostly NDMM samples (87.4%). Despite these differences, we were still able to identify and validate three prognostic genes.

The other esterases identified as being prognostic in our dataset, but not in the validation dataset, have all previously been reported to be dysregulated in cancer. *OVCA2* was originally identified as a tumour suppressor gene expressed in healthy surface epithelial cells of the ovary, and is downregulated in the majority of ovarian cell lines and tumours.^36^ *OVCA2* is located at chromosome 17p13, which is commonly deleted in haematological malignancies and also harbours several tumour suppressor genes, including *TP53*. In multiple myeloma, del17p is widely accepted to be one of the most aggressive features of the disease, and a marker of poor prognosis.^36–39^ In our dataset, OVCA2 was more highly expressed in RRMM versus NDMM samples, whereas in the MMRF CoMMpass dataset there was no difference in expression level. One possible explanation for this difference could be that our dataset had a lower frequency of del17p in RRMM samples (19/82, 23.2%) compared to the MMRF CoMMpass dataset (14/45, 31.1%). Also, in contrast with del17p being a marker of poor prognosis, our analysis of the FIMM dataset suggested that high expression of *OVCA2* is associated with poor prognosis in multiple myeloma, despite low expression of *OVCA2* being associated with del17p. Further research is required to determine the role of *OVCA2* in multiple myeloma. Upregulation of *PAFAH1B3* expression in multiple cancers has been observed previously,^4,40,41^ with selective inhibition impairing tumour cell survival.^42^ Very little has been published regarding *SIAE* in cancer cells. A recent in vitro study investigating the potential of targeting the ganglioside GD3 acetylation pathway to treat medulloblastoma showed that *SIAE* may be associated with mitochondria-mediated apoptosis and etoposide sensitivity.^43^ There are also very few publications on *PCED1B*. The role of *PCED1B* antisense RNA was investigated in an in vitro study of glioblastoma, which indicated that it functioned in a hypoxia-inducible factor (HIF)-1-dependent manner and has potential as a prognostic biomarker and druggable target for GBM.^44^ Further investigation into the role of these esterases in multiple myeloma may be warranted.

Our analysis did not take account of gene heterogeneity in CD138+ plasma cells, and the contribution of this heterogeneity to the observed differences in esterase gene expression. Further studies using single-cell RNA sequencing analysis would be useful in this regard. In RRMM patients, it is possible that prior treatment types also influenced the observed esterase gene expression patterns; this is again worthy of further study.

Interestingly, the frequency of SNVs appeared to be rare in the esterase genes, in both our dataset and the MMRF CoMMpass dataset. It has been previously reported that, except for a subset of specific genes, recurrent mutation rates are low in patients with multiple myeloma, suggesting that the dysregulation of key signalling pathways, rather than single-gene mutations, is the key driver for malignancy.^18,45^ Also, multiple myeloma is thought to be genetically heterogeneous, whereby clonal diversity would result in specific mutations only being present in a small number of cells within a tumour, confounding the molecular characterisation of tissue samples.^18^ However, in our study, several esterase genes were found to have a high frequency of duplication and deletion copy number alterations. To explore the possibility that tumours lacking SNVs might be driven by copy number alterations or chromosomal rearrangements, one study reported that of 153 patients with multiple myeloma, 119 (77.8%) had evidence of at least one focal gene copy number gain or loss within a significant peak, including 40 of 60 patients (66.6%) lacking somatic SNVs in the most significantly mutated genes.^18^ Further studies are required to establish the significance of esterase gene copy number alterations in patients with multiple myeloma.

One limitation of our expression analysis was the sample size; 123 samples were collected from patients with multiple myeloma, preventing statistically robust comparisons with the RRMM or NDMM samples. Another limitation was that matched samples were not widely available for patients progressing from NDMM to RRMM. Finally, regarding verification of the significance of esterase gene expression using the MMRF CoMMpass registry, it should be noted that this dataset was generated from diagnostic samples rather than treatment samples, and that the majority of the samples were taken from patients with NDMM; the possibility that some of our findings are more relevant to RRMM cannot be excluded.

In conclusion, specific esterases exhibited relatively high or low expression in multiple myeloma, and the esterase gene expression profile appeared to change on progression from NDMM to RRMM. High expression of *PAFAH1B3* and *SIAE*, and low expression of *PCED1B* were identified as poor prognostic markers in patients with multiple myeloma, suggesting a role for these esterases in myeloma biology. Further work is needed to elucidate the biological significance of esterases in cancer, to better understand how they can be effectively utilised to activate anticancer drugs in tumour cells, and their potential applicability as biomarkers of MM and its progression.

## Supplementary information

Supplemental material

## Data Availability

All data generated or analysed during this study are included in this article (and its supplementary information files).
