# Supplementary material for: Prognostic significance of esterase gene expression in multiple myeloma
Source: Br J Cancer. 2021 Feb 3;124(8):1428–36. doi: 10.1038/s41416-020-01237-1 (PMC8039029; doi:10.1038/s41416-020-01237-1)
Supplement: Supplementary file 1 — Supplemental material [file 41416_2020_1237_MOESM1_ESM.docx]

## Supplementary material:

# Prognostic significance of esterase gene expression in multiple myeloma

Romika Kumari, Muntasir Mamun Majumder, Juha Lievonen, Raija Silvennoinen, Pekka Anttila, Nina N. Nupponen, Fredrik Lehmann, and Caroline A. Heckman

**Corresponding author:** Dr. Caroline A. Heckman, Institute for Molecular Medicine Finland (FIMM), University of Helsinki, P.O. Box 20 (Tukholmankatu 8), 00014 University of Helsinki, Finland

**Email address:** caroline.heckman@helsinki.fi

### **SUPPLEMENTAL METHODS: DATA VALIDATION**

To further validate our results, clinical, gene expression and genomic variant data (somatic mutation and copy number variants [CNVs]) were obtained from the Multiple Myeloma Research Foundation (MMRF) Relating Clinical Outcomes in MM to Personal Assessment of Genetic Profile (CoMMpass) study (<https://research.themmrf.org> and [www.themmrf.org](http://www.themmrf.org)). The MMRF CoMMpass gene expression dataset included 892 samples in total: 875 bone marrow samples and 17 peripheral blood samples. The MMRF CoMMpass samples were obtained at baseline/diagnosis (*n* = 780), or in patients with progressive disease (*n* = 81), stable disease (*n* = 12), partial response (*n* = 7) and very good partial response (*n* = 4); for eight patients, disease information was missing. A total of 1044 samples (bone marrow *n* = 1021, peripheral blood *n* = 23) were used for CNV analysis; samples corresponded to baseline (*n* = 877), progressive disease (*n* = 90), partial response (*n* = 17), stable disease (*n* = 14), very good partial response (*n* = 9), complete response (*n* = 4) and stringent complete response (*n* = 1); information was missing in 32 samples. A total of 1164 samples (bone marrow *n* = 1140, peripheral blood *n* = 24) were included in the somatic mutation dataset; samples corresponded to baseline (*n* = 946), progressive disease (*n* = 122), stable disease (*n* = 20), partial response (*n* = 20), very good partial response (*n* = 16), and complete response (*n* = 6); information was missing in 34 samples.

#### ESTERASE GENES

#### Table S1. List of evaluated esterase genes (*N* = 51), sorted by median RPKM expression in 123 RNA-Seq samples from patients with multiple myeloma in the in-house FIMM cohort.

| **Gene** | **Genomic location** | **Median RPKM expression  (*N* = 123)** | **Full name** |
| --- | --- | --- | --- |
| *OVCA2* | 17p13.3 | 14.71 | Ovarian tumour suppressor candidate 2 |
| *PAFAH1B2* | 11q23.3 | 13.42 | Platelet-activating factor acetylhydrolase 1b catalytic subunit 2 |
| *NXPE3* | 3q12.3 | 10.22 | Neurexophilin and PC-esterase domain family member 3 |
| *UCHL3* | 13q22.2 | 7.61 | Ubiquitin C-terminal hydrolase L3 |
| *LIPA* | 10q23.31 | 7.52 | Lipase A, lysosomal acid type |
| *ABHD10* | 3q13.2 | 7.36 | Abhydrolase domain containing 10, depalmitoylase |
| *UCHL5* | 1q31.2 | 6.84 | Ubiquitin C-terminal hydrolase L5 |
| *CASD1* | 7q21.3 | 5.28 | CAS1 domain containing 1 |
| *ABHD13* | 13q33.3 | 4.66 | Abhydrolase domain containing 13 |
| *USP4* | 3p21.31 | 4.37 | Ubiquitin specific peptidase 4 |
| *IAH1* | 2p25.1 | 3.95 | Isoamyl acetate hydrolysing esterase 1 (putative) |
| *PCED1A* | 20p13 | 3.76 | PC-esterase domain containing 1A |
| *SIAE* | 11q24.2 | 3.32 | Sialic acid acetylesterase |
| *PON2* | 7q21.3 | 3.29 | Paraoxonase 2 |
| *NXPE4* | 11q23.2 | 3.04 | Neurexophilin and PC-esterase domain family member 4 |
| *ESD* | 13q14.2 | 2.50 | Esterase D |
| *PNPLA6* | 19p13.2 | 2.16 | Patatin like phospholipase domain containing 6 |
| *GZMA* | 5q11.2 | 2.04 | Granzyme A |
| *PCED1B* | 12q13.11 | 2.04 | PC-esterase domain containing 1B |
| *PAFAH1B3* | 19q13.2 | 1.91 | Platelet-activating factor acetylhydrolase 1b catalytic subunit 3 |
| *NCEH1* | 3q26.31 | 1.48 | Neutral cholesterol ester hydrolase 1 |
| *CPED1* | 7q31.31 | 1.31 | Cadherin like and PC-esterase domain containing 1 |
| *CES2* | 16q22.1 | 1.31 | Carboxylesterase 2 |
| *PAFAH2* | 1p36.11 | 1.30 | Platelet-activating factor acetylhydrolase 2 |
| *PNPLA4* | Xp22.31 | 1.23 | Patatin like phospholipase domain containing 4 |
| *GZMB* | 14q12 | 1.16 | Granzyme B |
| *GZMH* | 14q12 | 1.06 | Granzyme H |
| *CES4A* | 16q22.1 | 0.76 | Carboxylesterase 4A |
| *BPHL* | 6p25.2 | 0.59 | Biphenyl hydrolase like |
| *BCHE* | 3q26.1 | 0.53 | Butyrylcholinesterase |
| *UCHL1* | 4p13 | 0.45 | Ubiquitin C-terminal hydrolase L1 |
| *NXPE1* | 11q23.2 | 0.42 | Neurexophilin and PC-esterase domain family member 1 |
| *C1S* | 12p13.31 | 0.39 | Complement C1s |
| *NLGN4X* | Xp22.32-p22.31 | 0.30 | Neuroligin 4 X-linked |
| *PLA2G7* | 6p12.3 | 0.27 | Phospholipase A2 group VII |
| *CTLA4* | 2q33.2 | 0.21 | Cytotoxic T-lymphocyte associated protein 4 |
| *CES3* | 16q22.1 | 0.19 | Carboxylesterase 3 |
| *NLGN3* | Xq13.1 | 0.13 | Neuroligin 3 |
| *CES1* | 16q12.2 | 0.06 | Carboxylesterase 1 |
| *ACHE* | 7q22.1 | 0.05 | Acetylcholinesterase (Cartwright blood group) |
| *NXPE2* | 11q23.2-q23.3 | 0.04 | Neurexophilin and PC-esterase domain family member 2 |
| *NLGN1* | 3q26.31 | 0.03 | Neuroligin 1 |
| *NLGN2* | 17p13.1 | 0.03 | Neuroligin 2 |
| *PON1* | 7q21.3 | 0.02 | Paraoxonase 1 |
| *CEL* | 9q34.13 | 0.02 | Carboxyl ester lipase |
| *PON3* | 7q21.3 | 0.02 | Paraoxonase 3 |
| *NLGN4Y* | Yq11.221 | 0.01 | Neuroligin 4 Y-linked |
| *IL17A* | 6p12.2 | 0.00 | Interleukin 17A |
| *AADAC* | 3q25.1 | 0.00 | Arylacetamide deacetylase |
| *CES5A* | 16q12.2 | 0.00 | Carboxylesterase 5A |
| *ASPG* | 14q32.33 | 0.00 | Asparaginase |

*FIMM* Institute for Molecular Medicine Finland, *RNA-Seq* RNA sequencing, *RPKM* Reads Per Kilobase of transcript per Million mapped reads.

### **PATIENTS/SAMPLES**

#### Fig. S1. Flow chart for patients/samples included in the FIMM cohort analyses.


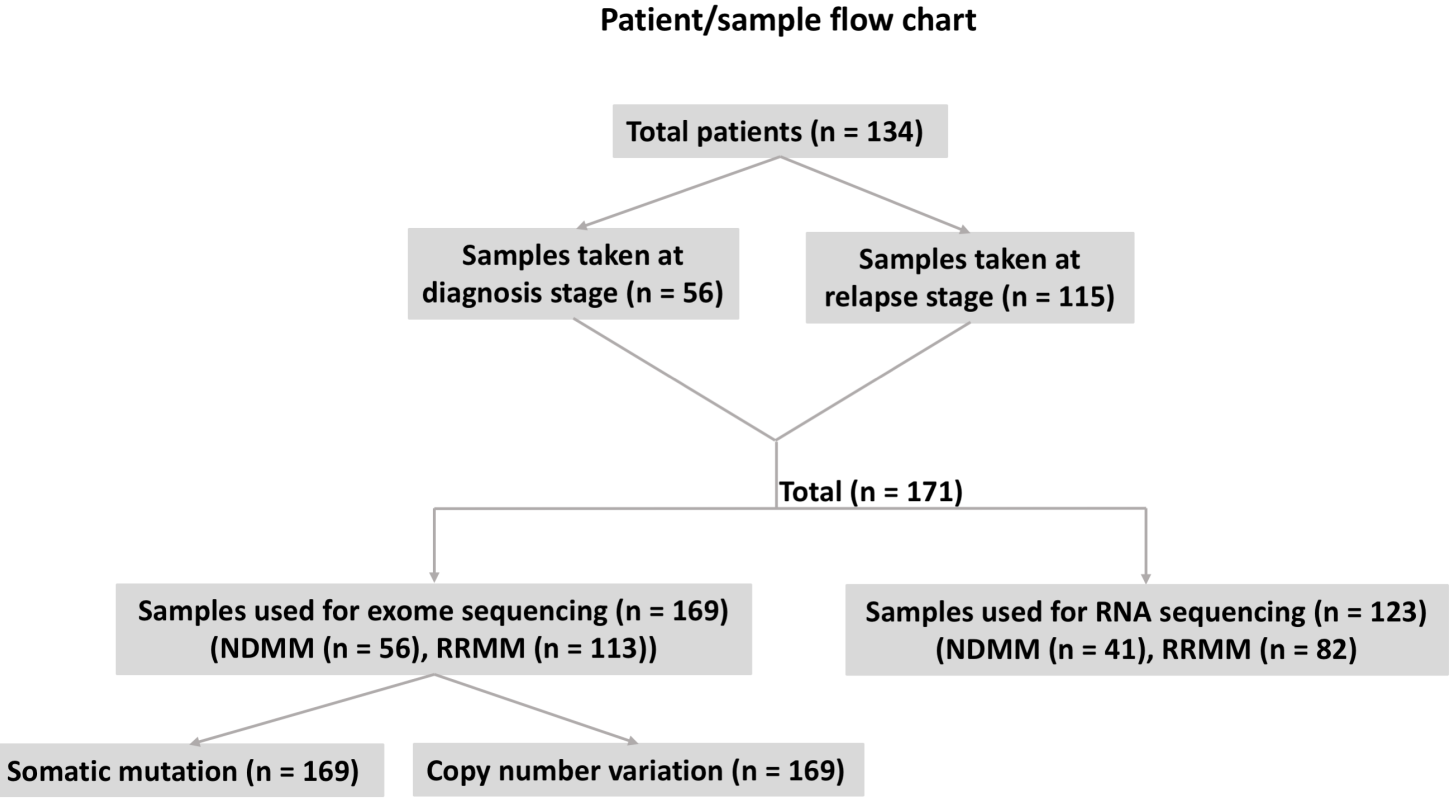


*FIMM* Institute for Molecular Medicine Finland, *NDMM* newly diagnosed multiple myeloma, *RRMM* relapsed/refractory multiple myeloma.

### **ESTERASE GENE EXPRESSION IN A LARGE MULTIPLE MYELOMA REGISTRY**

#### Fig. S2. Expression profile of esterase genes in the MMRF CoMMpass dataset (*N* = 892)^a^.


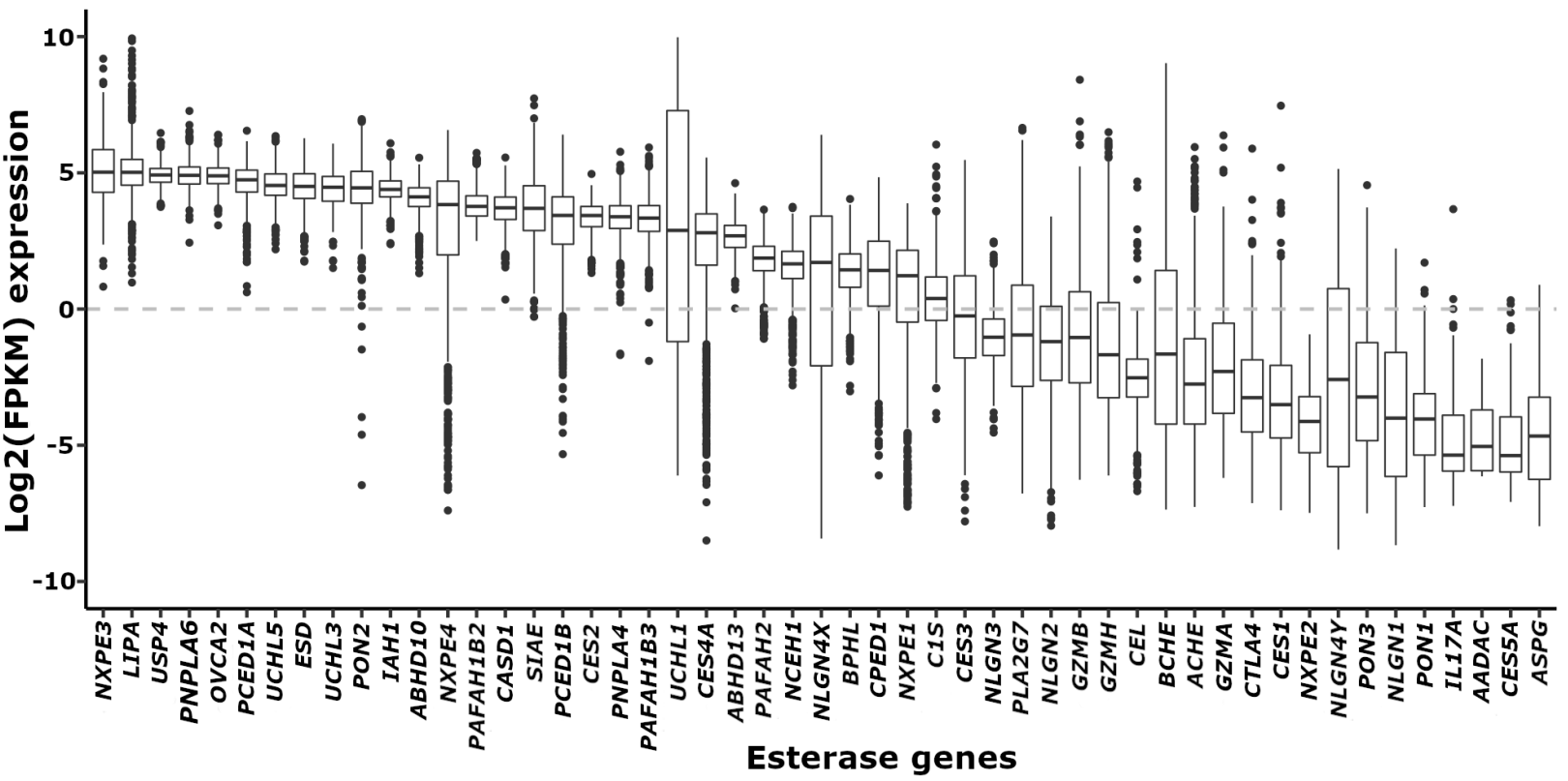


*CoMMpass* Relating Clinical Outcomes in MM to Personal Assessment of Genetic Profile study, *MMRF* Multiple Myeloma Research Foundation, *FPKM* Fragments Per Kilobase of transcript per Million mapped reads.

^a^Box plots: thick central line represents median; top and bottom lines of box represent third quartile and first quartile; whiskers indicate the variability in the data outside the upper and lower quartile; filled black circles represent outliers.

**Fig. S3.** The correlation in the esterase gene expression in FIMM and CoMMpass dataset, where each dot represents a gene (*n* = 51).


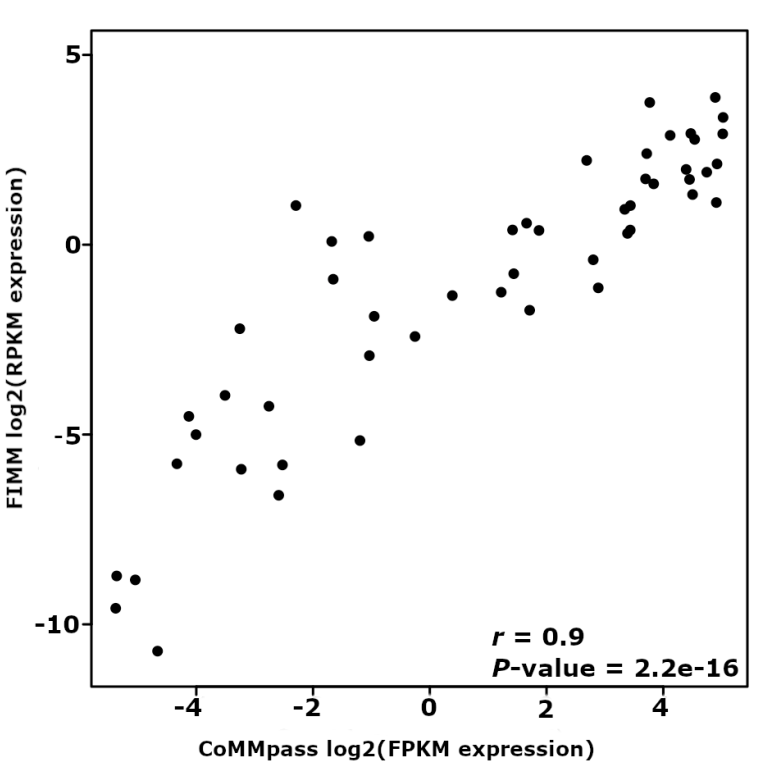


*CoMMpass* Relating Clinical Outcomes in MM to Personal Assessment of Genetic Profile study, *FIMM* Institute for Molecular Medicine Finland, *FPKM* Fragments Per Kilobase of transcript per Million mapped reads, *RPKM* Reads Per Kilobase of transcript per Million mapped reads.

#### Fig. S4. MMRF CoMMpass expression of esterases predicted in the FIMM cohort to have significant differential expression in NDMM (*n* = 39) versus RRMM (*n* = 45) (paired samples)^a^.


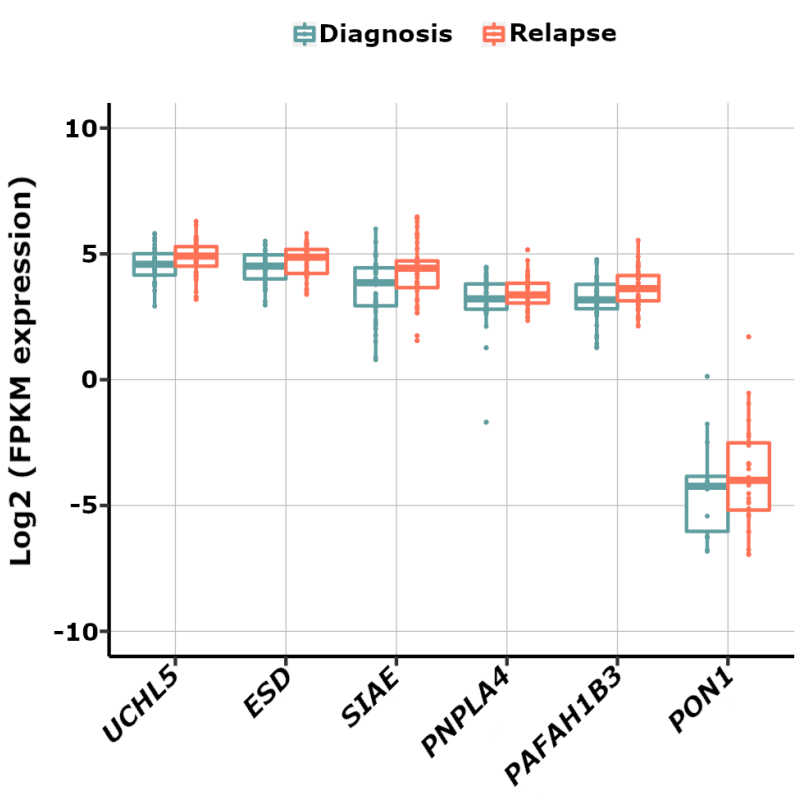


*CoMMpass* Relating Clinical Outcomes in MM to Personal Assessment of Genetic Profile study, *FIMM* Institute for Molecular Medicine Finland, *MMRF* Multiple Myeloma Research Foundation, *NDMM* newly diagnosed multiple myeloma, *FPKM* Fragments Per Kilobase of transcript per Million mapped reads, *RRMM* relapsed/refractory multiple myeloma.

^a^Box plots: thick central line represents median; top and bottom lines of box represent third quartile and first quartile; whiskers indicate the variability in the data outside the upper and lower quartile; circles inside the boxplot/distribution represent data point locations; circles outside the boxplot/distribution represent outliers.

**Fig. S5**. Esterase genes differential expression in samples with and without 1q gain. (A) The distribution of esterase log2(FC) and adjusted p values obtained in differential expression analysis of samples with 1q gain versus samples without 1q gain. (B) Expression distribution for the genes (n=4) found to be differentially regulated in the analysis.


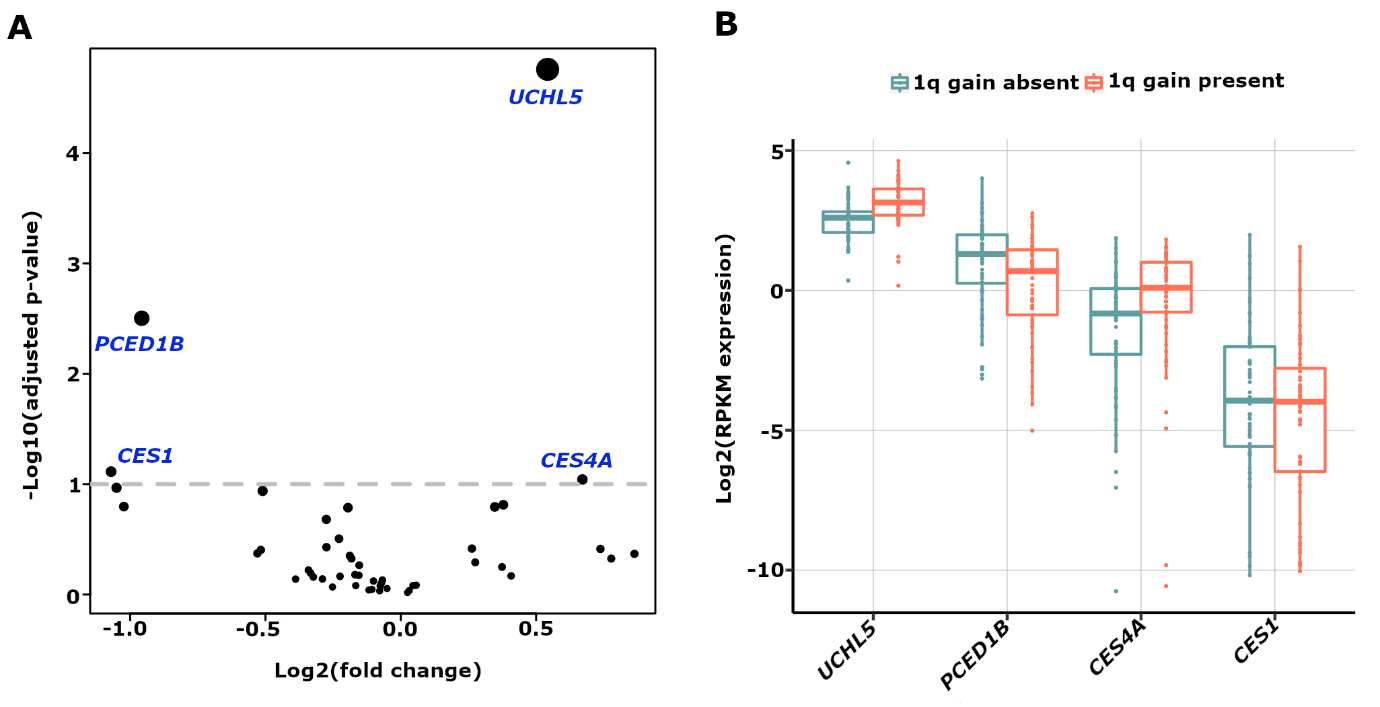


### **SURVIVAL ANALYSIS OF ESTERASES USING THE IN-HOUSE FIMM DATASET**

#### Table S2. Statistical overview for genes identified as prognostic markers (*P* ≤ 0.05) in the in-house FIMM dataset.

| **Gene** | ***P*-value** | **Median overall survival, months** | | **Hazard ratio (95% CL)** |
| --- | --- | --- | --- | --- |
|  |  | **High expression (95% CL)** | **Low expression (95% CL)** |  |
| *OVCA2* | <0.0001 | 68 (55–96) | 122 (100–NA) | 3.35 (1.811–6.198) |
| *PAFAH1B3* | 0.0042 | 73 (55–96) | 122 (100–NA) | 2.307 (1.31–4.062) |
| *GZMA* | 0.013 | 120 (94–127) | 55 (55–111) | 0.517 (0.306–0.874) |
| *PCED1B* | 0.026 | 100 (76–127) | 76 (55–122) | 0.546 (0.319–0.936) |
| *SIAE* | 0.027 | 68 (55–124) | 111 (89–142) | 1.87 (1.083–3.226) |
| *USP4* | 0.041 | 74 (55–111) | 122 (89–NA) | 1.76 (1.012–3.059) |
| *NXPE3* | 0.047 | 96 (76–127) | 76 (49–NA) | 0.578 (0.337–0.991) |

*CL* confidence limit, *FIMM* Institute for Molecular Medicine Finland, *NA* not available.

**Fig. S6.** Survival analysis log-rank test *P*-values from FIMM and CoMMpass dataset.


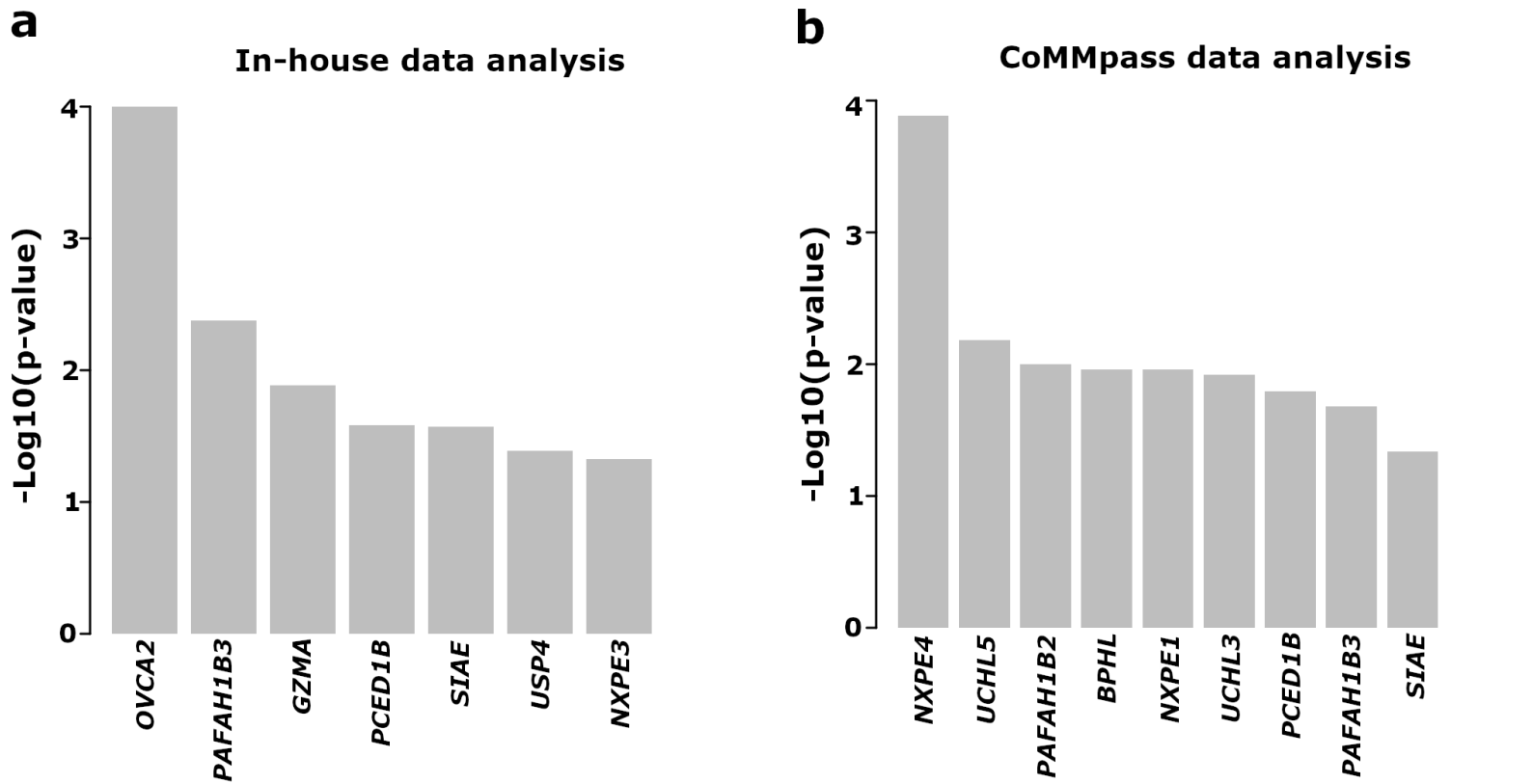


*CoMMpass* Relating Clinical Outcomes in MM to Personal Assessment of Genetic Profile study, *FIMM* Institute for Molecular Medicine Finland.

#### Fig. S7. Survival analysis of ‘other’ esterases identified as being prognostic in the in-house FIMM dataset, which were not validated in the MMRF CoMMpass dataset^a^.


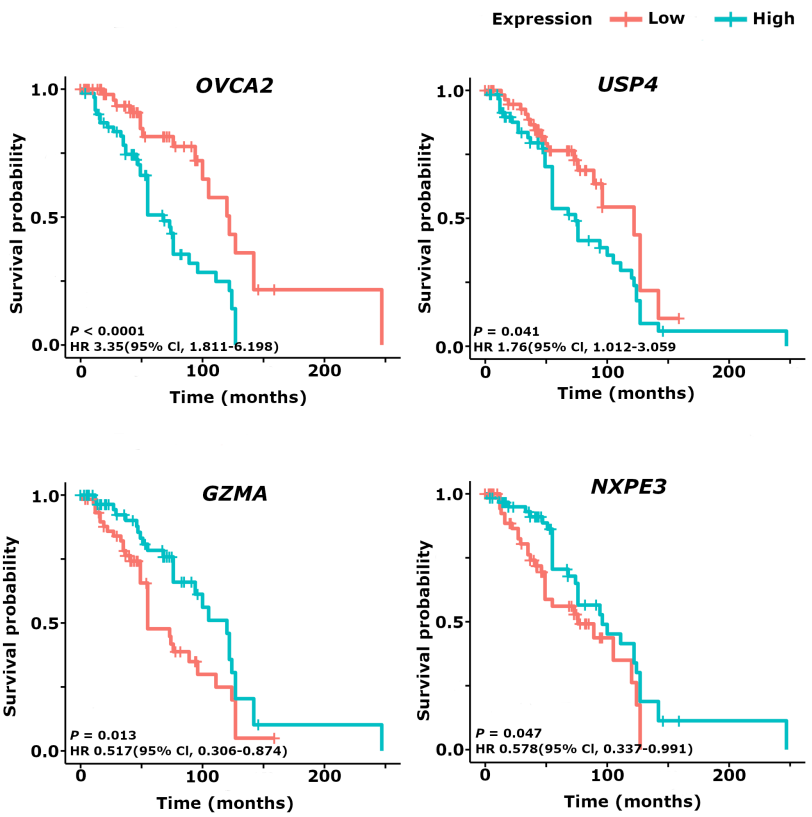


*CoMMpass* Relating Clinical Outcomes in MM to Personal Assessment of Genetic Profile study, *FIMM* Institute for Molecular Medicine Finland, *MMRF* Multiple Myeloma Research Foundation, *RPKM* Reads Per Kilobase of transcript per Million mapped reads.

^a^Only genes with RPKM expression >1 are included.

**Fig. S8**. Boxplot showing the comparison of gene OVCA2 expression which is located on 17p13.3 in the samples with and without del17p


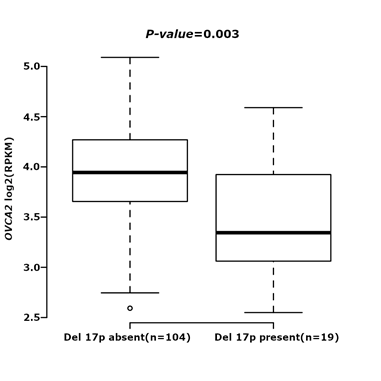


### **ESTERASE GENE SOMATIC MUTATION COPY NUMBER VARIATION IN THE FIMM AND MMRF COMMPASS DATASETS**

#### Fig. S9. Somatic single nucleotide variant frequency among esterase genes using exome sequencing data from 169 samples in the in-house FIMM cohort^a^.


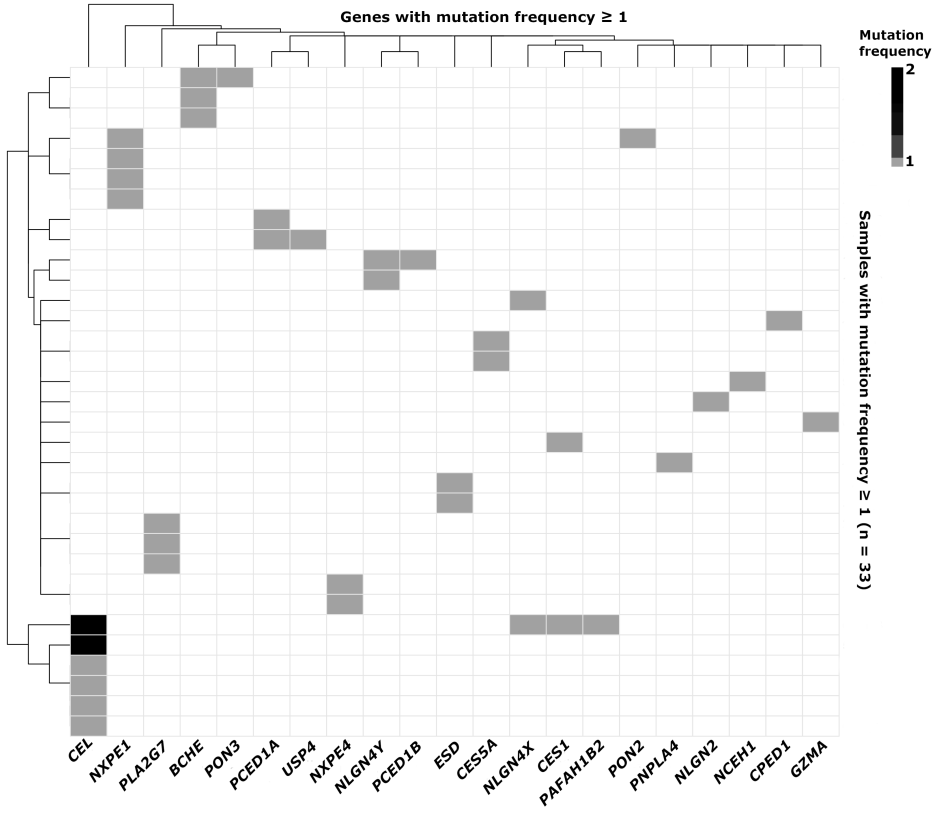


*FIMM* Institute for Molecular Medicine Finland.

^a^Only samples containing ≥1 esterase gene mutation are included in this figure (*n* = 33). Scale represents the total mutations predicted per gene per sample, where the maximum number of mutations recorded in a gene per sample is two (these mutations were present at different locations within the same gene).

#### Fig. S10. Somatic single nucleotide variant frequency among esterase genes using exome sequencing data from 1164 samples in the MMRF CoMMpass cohort^a^.


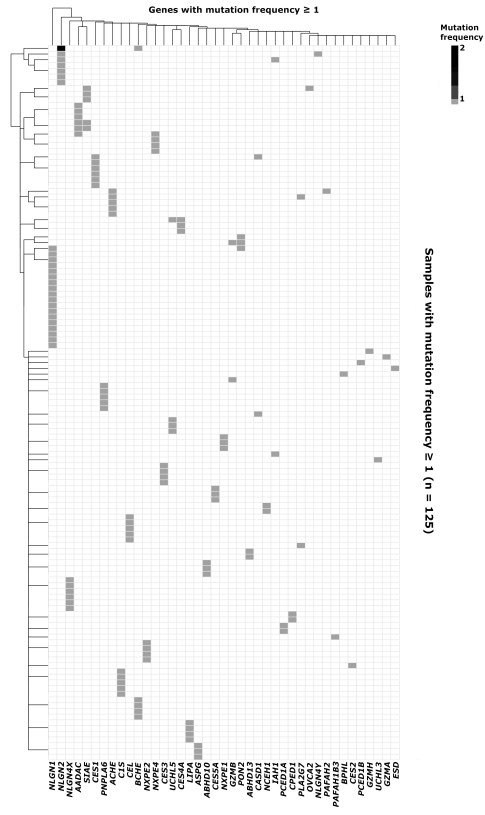


*CoMMpass* Relating Clinical Outcomes in MM to Personal Assessment of Genetic Profile study, *MMRF* Multiple Myeloma Research Foundation.

^a^Only samples containing ≥1 esterase gene mutation are included in this figure (*n* = 125). Scale represents the total mutations predicted per gene per samples, where the maximum number of mutations recorded in a gene per samples is two (these mutations were present at different locations within the same gene).

#### Fig. S11 (a) CNV heatmap clustering (based on CNV scores) from the MMRF CoMMpass dataset^a^. (b) Correlation of CNV percentages obtained from the FIMM and CoMMpass datasets for both gain and deletion variations.


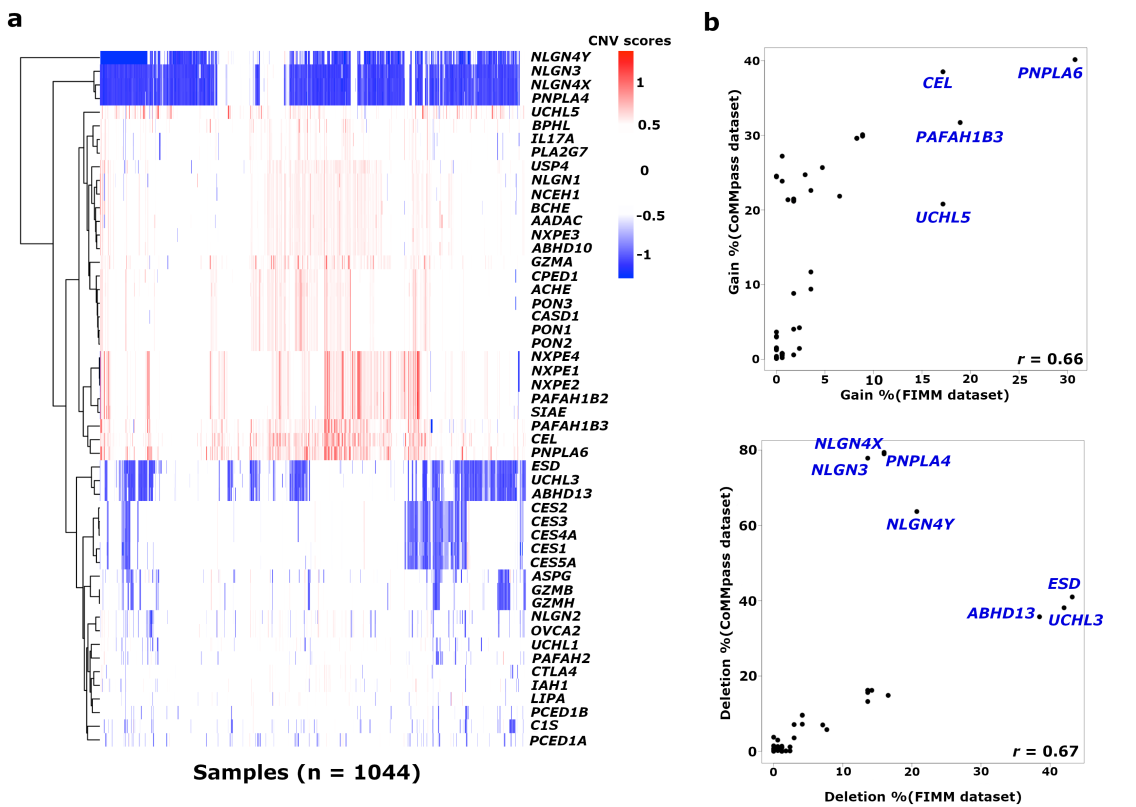


*CNV* copy number variation, *CoMMpass* Relating Clinical Outcomes in MM to Personal Assessment of Genetic Profile study, *FIMM* Institute for Molecular Medicine Finland, *MMRF* Multiple Myeloma Research Foundation.

^a^A CNV score of more than 0.5 predicts a duplication/gain event and a CNV score of less than −0.6 predicts a deletion event.
